# Annual Meetings of Hospitals

**Published:** 1921-04-23

**Authors:** 


### ANNUAL MEETINGS OF HOSPITALS.

### City of London Hospital for Diseases of the Chest.

Sir A. Kaye Butterworth, presiding at the annual Court of Governors at the Vintners' Hall, on April 4, said that, considering the difficulties with which hospitals are faced at the present time, the financial position may be regarded as fairly satisfactory. Mr. E. Jacobs complained that patients have to give the name of their employer, who, on being notified by the hospital, may think the employee is consumptive, and therefore dismiss him. An assurance was given that, in the event of any objection, the hospital would not insist on the information being given. The figures show there is an improvement in annual subscriptions, which amounted to £4,302, as against £4,054 in 1919, but the increase in income is mainly attributable to larger payments by the Insurance Committees and other public authorities, including the Ministry of Pensions, by whom a large number of ex-soldiers are referred for treatment. Since July last in-patients, with the exception of those sent by public authorities and necessitous cases, have been required to pay 10s. per week. Out-patients pay 1s. per attendance. The amount received last year from this source was nearly £800. The King's Fund made a grant on the usual lines of £2,500, and have promised £500 towards structural improvements. The King's Fund also made an emergency grant of £3,000, and £700 was received from the National Relief Fund. The Committee attribute to those exceptional receipts the gratifying fact that the hospital ended the year free from debt. The Committee of the Poplar Dispensary for the Prevention of Consumption, having made a gift of its equipment to the Borough Council's Tuberculosis Dispensary, transferred its hospital investments, to the value of £1,500, for the naming of a bed and cot, together with stocks and cash amounting to £220, for the creation of seven Life Governorships. The hospital is about to take over the land and buildings at Saunderton, Bucks, lent to the Invalid Children's Aid Association during the war, and propose making use of the twenty beds for convalescent women and children.

### The London Lock Hospital and Home.

Mr. J. F. W. Deacon, M.A., who at the March meeting was unanimously elected a Vice-President of the hospital, and is the representative of the Board on the City of Westminster Social Services Council, presided at the annual meeting held recently. The report states that the honorary staff of the hospitals decided at a special meeting of the Medical Committee to maintain the tradition of honorary service in connection with their work at the Harrow Road and Dean Street institutions, and the Board expresses the gratitude due to those members of the profession who make our voluntary system of hospitals possible. Under an arrangement with the L.C.C., paid clinical assistants, of whom four are qualified medical women, now attend at each out-patient clinic. On the suggestion of the Medical Committee it was decided to provide free facilities for teaching and post-graduate work to all members of the Fellowship of Medicine who apply for permission to attend the practice at the hospital. Babies born in the hospital free from venereal disease are now retained by their mothers when they enter the Home for training, and a special ward has been fitted up to meet the needs of this special class of case. Improvements in the machinery of the home laundry and structural alterations, etc., entailed the expenditure of some hundreds of pounds, and subscribers are earnestly asked to apply for the price-lists, as private laundry work can be done at rates which compare favourably with those of outside laundries.

